# Behind the Indolent Facade: Uncovering the Molecular Features and Malignancy Potential in Lung Minimally Invasive Adenocarcinoma by Single‐Cell Transcriptomics

**DOI:** 10.1002/advs.202303753

**Published:** 2023-11-22

**Authors:** Xin Zhang, Boxuan Liang, Yuji Huang, Hao Meng, Zhiming Li, Jiaxin Du, Lang Zhou, Yizhou Zhong, Bo Wang, Xi Lin, Guangchuang Yu, Xuewei Chen, Weixiang Lu, Zhe‐Sheng Chen, Xingfen Yang, Zhenlie Huang

**Affiliations:** ^1^ Department of Thoracic Surgery The First Affiliated Hospital of Guangzhou Medical University, State Key Laboratory of Respiratory Disease, National Clinical Research Center for Respiratory Disease, Guangzhou Institute of Respiratory Health Guangzhou 510140 China; ^2^ NMPA Key Laboratory for Safety Evaluation of Cosmetics Guangdong Provincial Key Laboratory of Tropical Disease Research School of Public Health Southern Medical University Guangzhou 510515 China; ^3^ Department of Bioinformatics School of Basic Medical Sciences Southern Medical University Guangzhou 510515 China; ^4^ College of Pharmacy and Health Sciences St. John's University Queens NY 11439 USA

**Keywords:** cathepsin B^+^ tumor‐associated macrophages, minimally invasive adenocarcinoma, senescent CD8^+^ T cells, single‐cell transcriptomics, tumor progression

## Abstract

The increased use of low‐dose computed tomography screening has led to more frequent detection of early stage lung tumors, including minimally invasive adenocarcinoma (MIA). To unravel the intricacies of tumor cells and the immune microenvironment in MIA, this study performs a comprehensive single‐cell transcriptomic analysis and profiles the transcriptomes of 156,447 cells from fresh paired MIA and invasive adenocarcinoma (IA) tumor samples, peripheral blood mononuclear cells, and adjacent normal tissue samples from three patients with synchronous multiple primary lung adenocarcinoma. This study highlights a connection and heterogeneity between the tumor ecosystem of MIA and IA. MIA tumor cells exhibited high expression of aquaporin‐1 and angiotensin II receptor type 2 and a basal‐like molecular character. Furthermore, it identifies that cathepsin B^+^ tumor‐associated macrophages may over‐activate CD8^+^ T cells in MIA, leading to an enrichment of granzyme K^+^ senescent CD8^+^ T cells, indicating the possibility of malignant progression behind the indolent appearance of MIA. These findings are further validated in 34 MIA and 35 IA samples by multiplexed immunofluorescence. These findings provide valuable insights into the mechanisms that maintain the indolent nature and prompt tumor progression of MIA and can be used to develop more effective therapeutic targets and strategies for MIA patients.

## Graphical abstract

This study uncovers the molecular features and malignancy potential in lung minimally invasive adenocarcinoma (MIA) by single‐cell transcriptomics. MIA tumor cells exhibit high expression of aquaporin‐1 and angiotensin II receptor type 2 and a basal‐like molecular character. Moreover, cathepsin B^+^ tumor‐associated macrophages overactivates CD8^+^ T cells, leading to an enrichment of granzyme K^+^ senescent CD8^+^ T cells in MIA.

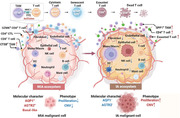

## 1 Introduction

Non‐small cell lung cancer constitutes 80% of all lung cancer cases, mostly adenocarcinoma subtype.^[^ ^1^ ^]^ The development of lung adenocarcinoma (LUAD) is believed to progress from adenocarcinoma in situ, to minimally invasive adenocarcinoma (MIA), and ultimately to fully invasive adenocarcinoma (IA).^[^ ^2^ ^]^ The increased use of low‐dose computed tomography screening has led to more frequent detection of early stage lung tumors, including T1mi (MIA) and T1a.^[^ ^3^ ^]^ However, a high detection rate also implies a risk of overdiagnosis. Moreover, MIA has been reported to have a more favorable prognosis than early stage invasive adenocarcinoma (T1a), even when their sizes are the same in both tumor types.^[^ ^4^ ^]^ This strengthens the clinical value of distinguishing MIA from IA for decision marking of surveillance and treatment strategies.

Given the low risk of recurrence and an almost complete 10‐year postoperative disease‐specific survival rate, surgical resection is considered a priority treatment option for MIA.^[^ ^5^ ^]^ However, because of the indolent growth pattern and good prognosis of MIA, a consensus has emerged that MIA should be treated less aggressively.^[^ ^6^, ^7^ ^]^ Indeed, recent advances in the development of targeted treatment and immunotherapy have accelerated progress in reducing lung cancer mortality.^[^ ^8^ ^]^ Yet, the efficacy of these therapies for MIA remains uncertain, and they are not currently the first choice for treating MIA.^[^ ^5^ ^]^ This underscores the urgency in decoding the molecular nature of MIA to provide rational support for these treatments. The lung tumor lesion comprises malignant cells, various types of immune cells, and stromal cells, forming a complex ecosystem.^[^ ^9^ ^]^ The heterogeneity of tumor cells and tumor microenvironment (TME) plays a vital role in shaping tumor behavior.^[^ ^10^, ^11^ ^]^ Thus, deciphering the complex interplay between tumor cells and the TME in MIA is of critical importance for a better understanding of the mechanisms that maintain the indolent nature and prompt tumor progression of MIA.

With the emergence of single‐cell RNA sequencing (scRNA‐seq), much effort has been devoted to elucidating the multicellular ecosystem of LUAD at different stages. While studies have revealed significant differences in the transcriptome of cancer cells and the tumor microenvironment (TME) between MIA and IA,^[^ ^12^, ^13^, ^14^, ^15^ ^]^ discrepancies in the findings persist due to the vast variability among patients. The main reason for this phenomenon is that the progression of LUAD is influenced by multiple factors, including genetic and epigenetic characteristics, environmental exposures, lifestyle habits, overall health, and more.^[^ ^1^, ^16^ ^]^ Therefore, it is crucial to eliminate these confounding variables to decode the indolent nature of MIA.

To unravel the intricacies of tumor cells and the immune microenvironment, while addressing the vast variation among patients, we conducted scRNA‐seq on fresh paired MIA and IA tumor samples, peripheral blood mononuclear cells (PBMC), and adjacent normal tissue samples (N) from three patients with synchronous multiple primary LUAD. We comprehensively identified the major cell types and analyzed the cell number composition of MIA compared to N and IA, as well as characterized transcriptome features and explored communication between different cell types. Furthermore, we validated our findings in an additional validation cohort using immunofluorescence (IF) staining. Our data sheds light on the unique aspects of the altered MIA tumor cell nature and TME and provides new insight into the biological basis of MIA and LUAD development. Our findings will thus help guide the diagnosis, surveillance, and treatment strategy of MIA.

## 2 Results

### 2.1 ScRNA‐seq Profiling of the Tumor and Immune Ecossystem in MIA and IA

**Figure 1:**
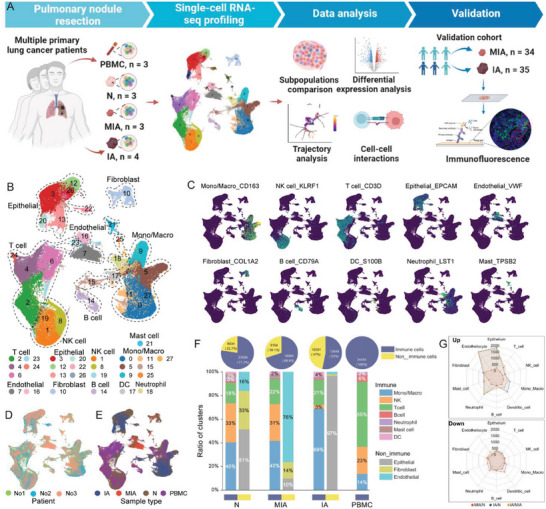
ScRNA‐seq profiling of the N, MIA, IA, and PBMC. A) Overview of the experimental design. B) UMAP plot showing the annotation and color codes for cell types in the lung tissues. C) UMAP plot showing the cell‐type annotation based on canonical marker gene expression. D) UMAP plot showing cell origins by patients. E) UMAP plot showing cell origins by sample types. F) Pie plots indicating the proportion of non‐immune and immune cells in each type of tissue (upper panel). Bar graph indicating the proportion of cells in each type of tissue. Non‐immune and immune cells are shown in separate bar graphs (lower panel). G) Radar plots showing the number of up‐regulated (upper panel) and down‐regulated (lower panel) DEGs in each cell type among the N, MIA, and IA tissues. DEGs, differentially expressed genes; IA, invasive adenocarcinoma; MIA, minimally invasive adenocarcinoma; N, adjacent normal tissue samples; PBMC, peripheral blood mononuclear cell; scRNA‐seq, single‐cell RNA sequencing; UMAP, uniform manifold approximation and projection.

We collected paired surgical MIA and IA specimens and N tissues from three treatment‐naive primary LUAD patients (discovery cohort) for scRNA‐seq. Specifically, there were 3 MIA samples, 4 IA samples, 3 non‐tumor tissues, and 3 PBMC samples collected and analyzed (Figure **1**A). Detailed clinical and pathological information, including tumor stage, and tumor size are provided in Table S1 (Supporting Information). After quality control, we analyzed 156447 cells in total (8789 to 15625 cells per sample with a mean of 12034 cells per sample). This generated over 7.7 billion total mapped reads and 23660 detected genes on average (Table S2, Supporting Information).

We performed cell classification and marker gene identification using Seurat to construct a panoramic tumor niche atlas. Our analysis identified 28 clusters, which we visualized using the uniform manifold approximation and projection (UMAP) method (Figure 1B). These cell clusters were then assigned to ten major known cell lineages according to the expression of canonical gene markers, including monocyte/macrophages, natural killer (NK) cells, T cells, epithelial cells, endothelial cells, fibroblasts, B cells, dendritic cells (DC), neutrophils, and mast cells (Figure 1B,C; Figure S1, Suppporting Information). It is noteworthy that all these cell subtypes were shared consistently across patients as well as within the IA, MIA, N, and PBMC samples, albeit with variations in their relative proportions (Figure 1D–F). The proportions of non‐immune cells, primarily consisting of epithelial cell adhesion molecule^+^ (EpCAM^+^) epithelial cells, endothelial cells, and fibroblasts, increased sequentially from non‐tumor tissues to MIA and IA samples. Notably, the proportions of these cell types were diverse in the three groups. The non‐immune cells in MIA were predominantly endothelial cells (76%), while in IA they were mostly malignant epithelial cells (97%). Conversely, the proportions of immune cells in MIA and N were comparably distributed, primarily encompassing monocytes/macrophages, NK cells, and T cells. However, within IA, monocyte/macrophage proportions surpassed the former two, whereas NK cell proportions were reduced. Furthermore, in contrast, PBMC demonstrated a distinctive composition of immune cells, significantly divergent from the preceding three tissues, predominantly comprising T cells (Figure 1F; Table S3, Supporting Information).

To gain insight into the molecular variation among the different stages of LUAD, we conducted a comparison of gene expression profiles among the N, MIA, and IA tissues (Figure 1G). The number of differentially expressed genes (DEGs) between MIA and N was significantly lower when compared to the number of DEGs between IA and MIA, as well as between IA and N. This suggests that the gene expression profile of MIA is more like that of N than that of IA. Moreover, when compared to N, the up‐regulated DEGs in IA were predominantly found in epithelial and endothelial cells, while the up‐regulated DEGs in MIA were found primarily in fibroblasts. Our results suggest that MIA represents a similar immune ecosystem compared to N but has a distinct tumor and immune ecosystem compared to IA.

### 2.2 Hallmark Signatures for Malignant Cells of MIA

**Figure 2:**
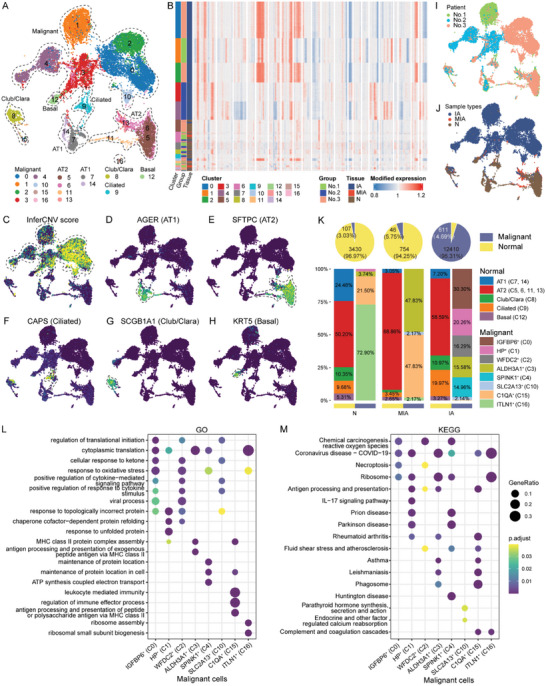
Subtypes of epithelial cells in N, MIA, and IA. A) UMAP plot displaying the subtypes of epithelial cells in the lung tissues, color‐coded based on cell type annotation. B) Heatmap showing large‐scale inferCNVs in cluster order for individual epithelial cells in N, MIA, and IA. C) UMAP plot showing the inferCNV score in each cell. D–H) UMAP plot showing the cell‐type annotation based on canonical marker gene expression. I) UMAP plot showing cell origins by patients. J) UMAP plot showing cell origins by sample types. K) Pie plots depicting the proportion of normal and malignant epithelial cells in each sample type (upper panel); bar graph indicating the proportion of subclusters in each sample type, with separate bar graphs for normal and malignant epithelial cells (lower panel). L) Bubble chart showing the enrichment of biological processes in GO analysis. M) Bubble chart showing the enrichment of pathways in KEGG analysis. *AGER*, advanced glycosylation end‐product specific receptor; AT1, alveolar type I cells; AT2, alveolar type II cells; *CAPS*, calcyphosine; CNV, copy number variant; GO, Gene Ontology; IA, invasive adenocarcinoma; KEGG, Kyoto Encyclopedia of Genes and Genomes; *KRT5*, keratin 5; MIA, minimally invasive adenocarcinoma; N, adjacent normal tissue samples; *SCGB1A1*, secretoglobin family 1A member 1; *SFTPC*, surfactant protein C; UMAP, uniform manifold approximation and projection.

Next, we focused on the transcriptomic features of epithelial cells in N, MIA, and IA tissues. Among the 17380 epithelial cells, we identified 17 sub‐clusters by unsupervised clustering method and visualized using the UMAP method (Figure **2**A). We separate eight malignant cell clusters (C0‐4, C10, C15 and C16) from normal cell clusters by comparing their copy number variants (CNVs) using the inferCNV^[^ ^17^ ^]^ method (Figure 2B,C). Each malignant cell cluster was named by its top positive marker gene (Figure S2A, Supporting Information). Particularly, we found low CNV in the MIA tissues (Figure S2B, Supporting Information), indicating that the MIA tissues have not fully exhibited malignant characteristics. We then categorized the normal cell clusters into five distinct normal cell types based on their canonical markers: alveolar type I cells (AT1), club and clara cells (Club/Clara), alveolar type II cells (AT2), ciliated airway epithelial cells (Ciliated), and basal cells (Basal) (Figure 2D–H; Figure S2C, Supporting Information). It is noteworthy that the normal cell clusters shared across patients, while the malignant cell clusters variant across patients, underlining pronounced interpatient heterogeneity in malignant cells rather than normal cells (Figure 2C,I). Notably, we discerned that cells originating from MIA are interspersed among cells from normal tissue, while being clearly distinct from cells originating from IA (Figure 2J). This observation suggests that the transcriptomic characteristics of cells derived from MIA are more similar to those of cells from normal tissue, as opposed to cells originating from IA.

As the progression of LUAD advanced, the proportion of malignant cells escalated (Figure 2K). Despite the relatively limited number of malignant cells, those from MIA were primarily comprised of two main clusters: aldehyde dehydrogenase 3 family member A1^+^ (ALDH3A1^+^, C3) and complement C1q A chain^+^ (C1QA^+^, C15) cells. Gene Ontology (GO) and Kyoto Encyclopedia of Genes and Genomes (KEGG) analysis indicated these clusters' involvement in antigen processing and presentation (Figure 2L,M). In stark contrast, no dominant malignant cell clusters emerged within those originating from IA. Furthermore, the biological processes and signaling pathways exhibited notable variations across these distinct malignant cell clusters (Figure 2L,M).

**Figure 3:**
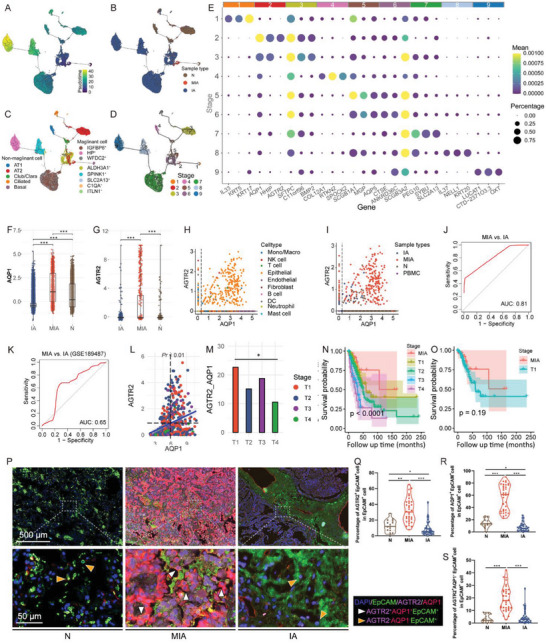
Analysis of epithelial cell transition states in N, MIA, and IA samples. Pseudotime‐ordered analysis of epithelial cells from N, MIA, and IA samples, cells are colored by A) pseudotime, B) sample type, C) cell type, and D) transition states. E) Heatmap indicating the expression of marker genes in each transition states of epithelial cells. Bar plots indicating the expression of F) *AQP1* and G) *AGTR2* in each sample type. Scatter diagram showing the expression of *AGTR2* and *AQP1* in each cell, colored by H) cell type and I) sample type. ROC curves of *AGTR2* and *AQP1* for distinguishing MIA and IA in J) our dataset and K) in GSE189487. L) Scatter diagram showing the positive correlation between expression of *AGTR2* and *AQP1* in TCGA‐LUAD dataset. M) Bar plot showing the proportion of patients with high expression of both *AGTR2* and *AQP1* in each cancer stage in TCGA‐LUAD dataset. N) Kaplan‐Meier analysis showing the relapse‐free survival rate of all LUAD patients, characterized by cancer stages. Significance was calculated using the log‐rank test. O) Kaplan‐Meier analysis showing the relapse‐free survival rate between MIA and T1 stage of LUAD. Significance was calculated using the log‐rank test. P) Representative image of IF staining for AGTR2, AQP1, and EpCAM, showing the proportion of AQP1^+^ AGTR2^+^ epithelial cells in epithelial cells in N, MIA, and IA samples from the discovery cohort. Violin plot illustrating the proportion of Q) AQP1^+^, R) AGTR2^+^ and S) AQP1^+^AGTR2^+^ epithelial cells in epithelial cells in N, MIA, and IA samples from the validation cohort, based on IF results. Statistical analyses are determined by Wilcoxon test. ^*^ *p* < 0.05, ^**^ *p* < 0.01, ^***^ *p* < 0.001. *AGTR2*, angiotensin II receptor type 2; *AQP1*, aquaporin‐1; IA, invasive adenocarcinoma; LUAD, lung adenocarcinoma; MIA, minimally invasive adenocarcinoma; N, adjacent normal tissue samples; TCGA, The Cancer Genome Atlas.

Next, we investigated the dynamic states and transitions in the epithelial cells by utilizing Monocle3. The analysis revealed that the malignant cells might arise from AT1, AT2, and basal cells. These three cell types, despite having distinct origins, share a similar trajectory path during the later process of malignancy (Figure **3**A–C). Notably, we found an intermediate cell state positioned between basal cells and malignant cells, exclusively comprised of cells originating from MIA (Figure 3B,C). This subcluster comprises cells from different patients, thereby excluding the potential influence of interpatient heterogeneity on the trajectory (Figure S3A, Supporting Information). Subsequently, we employed the slingshot^[^ ^18^ ^]^ and scTour^[^ ^19^ ^]^ method to infer the state trajectories, which yielded similar paths and developmental dynamics (Figure S3B,C, Supporting Information), affirming the robustness of the results obtained through Monocle3. Our findings highlight a distinctive cell state within MIA epithelial cells and implies a basal‐like molecular nature for a predominant subset of pre‐malignant epithelial cells in MIA.

We divide the epithelial cells into nine stages according to their pseudotime ordering (Figure 3D). By using the “‘FindAllMarkers”’ function in Monocle3, we identified both aquaporin‐1 (AQP1) and angiotensin II receptor type 2 (AGTR2) as distinctive markers associated with the MIA‐specific stage (stage 2, Figure 3E; Figure S2, Supporting Information). The expression of these two genes was significantly elevated in MIA epithelial cells compared to those in IA and N (Figure 3F,G). To explore whether *AQP1* and *AGTR2* may server as MIA biomarkers in bulk‐level RNA‐seq data, we investigated their expression across all cell types in our scRNA‐seq data. While *AQP1* and *AGTR2* exhibited expression in other cell types (Figure S3D,E, Supporting Information), we observed that nearly all cells with both high *AQP1* and *AGTR2* expression belonged to the epithelial (Figure 3H). Furthermore, this high expression was predominantly observed in cells from MIA samples (Figure 3I), and the combination of *AQP1* and *AGTR2* efficiently discriminated MIA cells from IA (Figure 3J). This was corroborated using a publicly available scRNA‐seq data^[^ ^14^ ^]^ (Figure 3K). Our results indicates that the combination of *AQP1* and *AGTR2* may serve as epithelial cell‐specific MIA biomarkers.

Subsequently, we sought to apply these two markers to the LUAD cohort from The Cancer Genome Atlas (TCGA) database to distinguish lung cancer patients with MIA features from others. While the observed differences lack statistical significance, we observed a gradual reduction in the expression of both *AQP1* and *AGTR2* as tumor progression advanced (Figure S3F,G, Supporting Information). These two markers were co‐expressed in pairs and showed positive correlations in their expression levels (Figure 3L). Importantly, the proportion of *AQP1* ^high^ *AGTR2* ^high^ patients was significantly higher in the T1 stages compared to the late stage (T4) of LUAD (Figure 3M), indicating that the combination of *AQP1* and *AGTR2* have a high capacity for distinguishing early from late stage of LUAD patients. Furthermore, we defined patients in the T1 stage with AQP1^high^AGTR2^high^ as patients with MIA features and found that they had a higher probability of survival compared to patients in other stages (Figure 3N,O). Using IF staining, we measured the proportion of AQP1^+^ and AGTR2^+^ cells in EPCAM^+^ cells in the tissues of both discovery and validation cohorts, where we found that the proportion of AQP1^+^, AGTR2^+^, and AQP1^+^AGTR2^+^cells in EPCAM^+^ cells was significantly higher than that in N and IA in both cohorts (Figure 3P–S). Taken together, our findings identify the combination of AQP1 and AGTR2 as hallmark signatures of malignant cells in MIA, enabling precise distinction of MIA from IA.

### 2.3 Dysfunctional Granzyme K (GZMK)^+^ CD8^+^ T Cells Abundant in MIA

**Figure 4:**
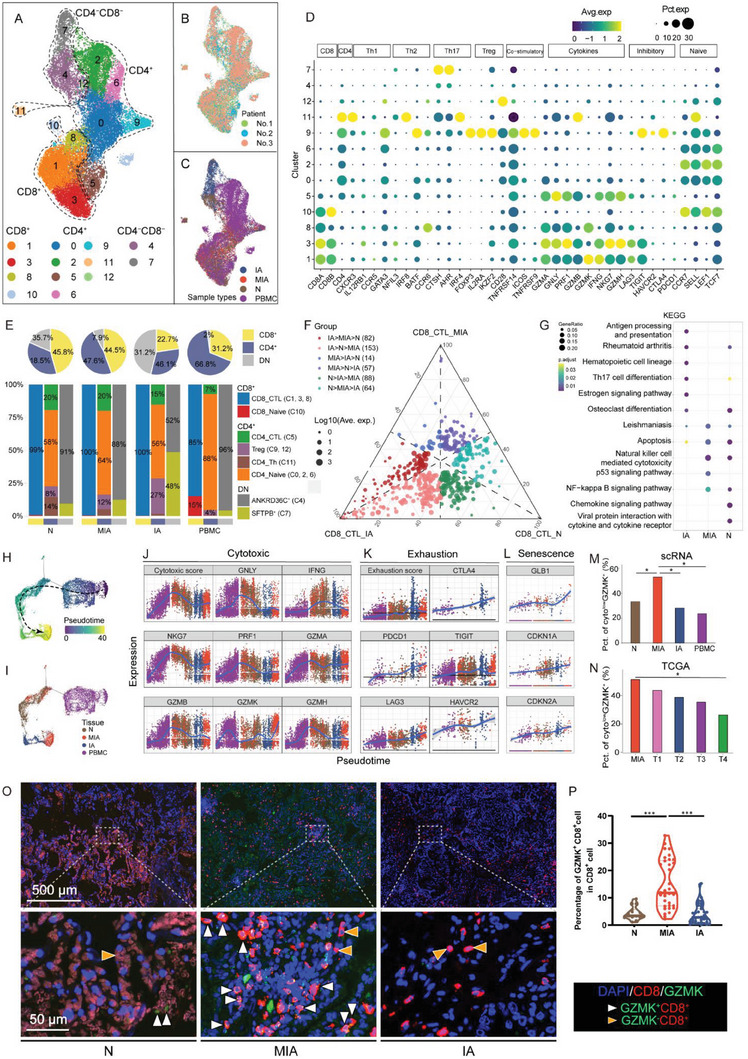
T cell subtypes in N, MIA, IA, and PBMC. A) UMAP plot showing the annotation and color codes for the subtypes of T cells in N, MIA, IA, and PBMC. B) UMAP plot showing cell origins by patients. C) UMAP plot showing cell origins by sample types. D) UMAP plot showing the expression of selected gene sets in each subcluster of T cells. E) Pie plots indicating the proportion of CD4^+^, CD8^+^, and DN T cells in each type of tissues (upper panel). Bar graph indicating the proportion of subclusters in each type of tissues. CD4^+^, CD8^+^, and DN T cells are shown in separate Bar graphs (lower panel). F) Ternary diagram showing the DEGs of CD8^+^ CTL among N, MIA, and IA tissues. G) Enrichment of KEGG pathways for high expressed DEGs in N, MIA, and IA tissues. Pseudotime‐ordered analysis of CD8^+^ T cells from N, MIA, and IA samples, cells are colored by H) pseudotime, and I) sample type. 2D plots showing the J) cytotoxic score and the expression of relative genes, K) exhaustion score and the expression of relative genes, and L) the expression of senescent genes in each sample type, along with the pseudotime. M) Scatter diagram showing the positive correlation between expression of *GZMK* and cytotoxic score in TCGA‐LUAD dataset. N) Bar plot in the showing the proportion of patients with high expression of GZMK and low cytotoxic score in each cancer stage in TCGA‐LUAD dataset. O) Representative image of IF staining for GZMK and CD8, showing the proportion of GZMK^+^ CD8^+^ T cells in T cells in N, MIA, and IA samples from discovery cohort. P) Violin plot illustrating the proportion of GZMK^+^ CD8^+^ T cells in T cells in N, MIA, and IA samples from validation cohort, based on IF results. Statistical analyses are determined by Wilcoxon test. ^*^ *p* < 0.05, ^**^ *p* < 0.01, ^***^ *p* < 0.001. DEG, differentially expressed gene; DN, double negative; GZMK, granzyme K; IA, invasive adenocarcinoma; KEGG, Kyoto Encyclopedia of Genes and Genomes; LUAD, lung adenocarcinoma; MIA, minimally invasive adenocarcinoma; N, adjacent normal tissue samples; PBMC, peripheral blood mononuclear cell; TCGA, The Cancer Genome Atlas; UMAP, uniform manifold approximation and projection.

We performed unsupervised clustering on 26 079 T cells, resulting in the identification of 13 sub‐clusters (Figure **4**A). All these cell clusters were shared consistently across patients as well as within the IA, MIA, N, and PBMC samples (Figure 4B,C). Among the 26 079 T cells, we identified four clusters for CD8^+^ T cells (C1, C3, C8, and C10), seven clusters for CD4^+^ T cells (C0, C2, C5, C6, C9, C11, and C12), and two cluster (C4 and C7) for double negative (DN) T cells (Figure 4A,D). We characterized the CD8^+^ T cell subtypes as cytotoxic (C1, C3, and C8) and naive (C10) based on their cytokine expression and naive markers. The CD4^+^ T cell subtypes were characterized as cytotoxic (C5), naive (C0, C2, and C6), and Treg (C9 and C12), T helper (C11) cells, based on their expression of canonical markers (Figure 4D). Additionally, we assigned names to the two DN clusters based on their top positive marker genes: ankyrin repeat domain 36C (*ANKRD36C*) and surfactant protein B (*SFTPB*), respectively (Figure S4A, Supporting Information). GO and KEGG analyses shows distinct characteristics and features of these cell subclusters (Figure S4B,C, Supporting Information). The proportion of CD4^+^ naive T cells in MIA was higher than that in IA and N, while the proportion of CD4^+^ Th cells was lower. The proportion of SFTPB^+^ DN T cells (C7) was higher in IA compared to MIA and N, whereas the proportion of ANKRD36C^+^ DN T cells (C4) in MIA was higher compared to IA but similar to N. Additionally, the proportion of Treg cells increased along with tumor progression (Figure 4E). Our results suggest that LUAD microenvironment lacks mature and functional CD4^+^ T cells at the pre‐malignant stage of MIA. Moreover, PBMC displayed a distinct composition of CD4^+^ T cell subpopulation, differing significantly from that in the previous three tissues (Figure 4E).

The proportions of CD8^+^ T cells in MIA samples were comparable to those in normal samples, while the proportions of CD8^+^ T cells in IA samples were lower than those in both MIA and normal samples. Additionally, the proportion of CD4^+^ cytotoxic T cells in IA samples was also lower than that in both MIA and normal samples (Figure 4E), indicating that the microenvironment of MIA has a sufficient cytotoxic capacity, while the microenvironment of IA has an exhausted cytotoxic capacity. To further examine the transcriptional features of CD8^+^ T cells in MIA and IA samples, we compared the gene expression of CD8^+^ cytotoxic T lymphocytes (CTLs) in normal, MIA, and IA samples. A DEG analysis revealed that many genes were up‐regulated in IA when compared to MIA and normal, while only a limited number of genes were up‐regulated in MIA when compared to IA and normal (Figure 4F). A KEGG analysis showed that CD8^+^ CTLs in MIA were characterized by an upregulation of the cytotoxicity, P53, and NF‐κB signaling pathways, indicating that these cells were functioning well in the MIA microenvironment (Figure 4G).

The pseudotime analysis showed that CD8^+^ T cells originated from naive CD8^+^ T cells (Figure S4D, Supporting Information), sharing the same transition trajectory but residing in different states in the PBMC, N, MIA, and IA samples (Figure 4H,I). The CD8^+^ T cells in PBMC were at the start of the trajectory, followed by a portion of CD8^+^ T cells from MIA and all the CD8^+^ T cells from N. Meanwhile, the CD8^+^ T cells in IA were in a late state, while some CD8^+^ T cells in MIA were at the end of the trajectory (Figure 4I). Slingshot^[^ ^18^ ^]^ method yielded similar trajectories, affirming the robustness of the results obtained through Monocle3 (Figure S4E, Supporting Information). We found that the cells in each transition state come from different patients, indicating limited affection of interpatient heterogeneity on the trajectory (Figure S4F, Supporting Information). The cytotoxic and exhausted scores showed that the transition started with naive CD8^+^ T cells, then proceeded through an intermediate state characterized by high cytotoxicity and low exhaustion, before finally reaching an exhausted state with high exhaustion and low cytotoxicity. Interestingly, we found that the expression of *GZMK*, a cytotoxic marker, did not increase until the end of trajectories (Figure 4J,K). This suggests the presence of a specific senescent subset of CD8^+^ T cells in MIA with low cytotoxicity but high *GZMK* expression (Cyto^low^GZMK^+^). Indeed, we observed elevated expression of senescence markers including galactosidase beta 1 (*GLB1*), cyclin dependent kinase inhibitor 1A (*CDKN1A*), and cyclin dependent kinase inhibitor 2A (*CDKN2A*) in this subset (Figure 4L). Furthermore, we found that the proportion of Cyto^low^GZMK^+^ CD8^+^ T cell in MIA was significantly higher than those in N, IA, and PBMC (Figure 4M). We confirmed this finding in the TCGA database, where the proportion of Cyto^low^GZMK^+^ patients in MIA‐like stage was higher than that in other stages (Figure 4N). Using IF, we measured the proportion of GZMK^+^ cells in CD8^+^ T cells in the tissues of both discovery and validation cohorts, where we confirmed the abundance of a subset of GZMK^+^ CD8^+^ T cells in MIA, but not in N and IA (Figure 4O,P). Taken together, both the number and cytotoxic capacity of CD8^+^ T cells decreased as the LUAD progressed. The CD8^+^ T cells in MIA were comprised of both a functional T cells and a dysfunctional low cytotoxic GZMK^+^ senescent subset. This may partially explain why MIA has limited progression, but also highlights the potential risk for lacking a reserve of functional CD8^+^ T cells.

### 2.4 Cathepsin B (CTSB)+ Tumor‐Associated Macrophages (TAMs) as the Early Responder in the MIA Stage of LUAD

**Figure 5:**
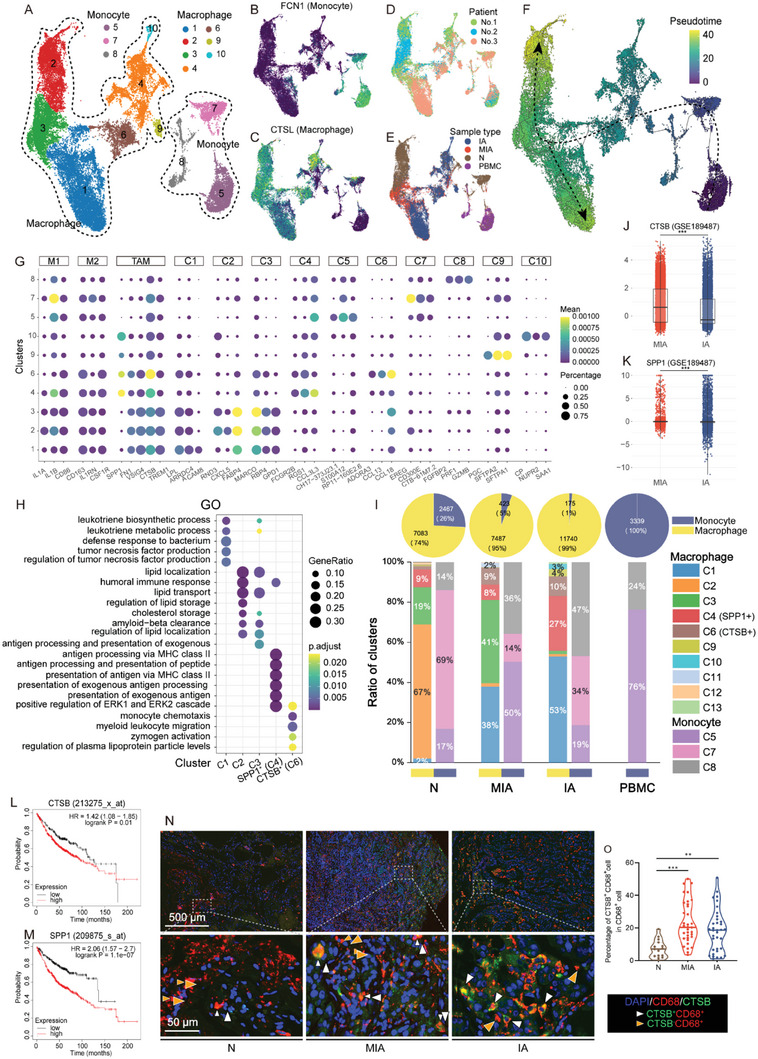
Subtypes of monocytes and macrophages in N, MIA, IA, and PBMC. A) UMAP plot showing the annotation and color codes for the subtypes of monocyte and macrophages in N, MIA, IA, and PBMC. B) UMAP plot showing the cell‐type annotation based on the canonical marker gene expression. Pseudotime‐ordered analysis of monocyte and macrophages from N, MIA, IA, and PBMC samples, cells are colored by C) pseudotime, D) sample type, E) Seurat subclusters, and F) transition states. G) Dot plot showing the expression of canonical marker gene of M1, M2, and TAM and the marker genes in each transition states of monocyte and macrophages. H) Enrichment of GO for the marker genes of each transition states. I) Pie plots indicating the proportion of monocyte and macrophages in each type of tissue (upper panel). Bar graph indicating the proportion of subclusters in each type of tissues. monocyte and macrophages are shown in separate bar graphs (lower panel). Bar plots indicating the expression of J) *CTSB* and K) *SPP1* in MIA and IA samples in GSE189487 dataset. Kaplan‐Meier analysis showing the relapse‐free survival rate of all LUAD patients in Gyorffy’ dataset, characterized by either low or high expression of L) *CTSB* and M) *SPP1*. N) Representative image of IF staining for CTSB and CD68, showing the proportion of CTSB^+^ CD68^+^ macrophages in CD68^+^ macrophages in N, MIA, and IA samples from discovery cohort. The white arrowheads indicate CTSB^+^ CD68^+^ macrophages and the yellow arrowheads indicate CTSB^−^ CD68^+^ macrophages. O) Violin plot illustrating the proportion of CTSB^+^ CD68^+^ macrophages in CD68^+^ macrophages in N, MIA, and IA samples from the validation cohort, based on IF results. Statistical analyses are determined by Wilcoxon test. ^*^ *p* < 0.05, ^**^ *p* < 0.01, ^***^ *p* < 0.001. *CTSB*, cathepsin B; GO, Gene Ontology; IA, invasive adenocarcinoma; LUAD, lung adenocarcinoma; MIA, minimally invasive adenocarcinoma; N, adjacent normal tissue samples; PBMC, peripheral blood mononuclear cell; *SPP1*, secreted phosphoprotein 1; UMAP, uniform manifold approximation and projection.

Our data indicates that monocytes/macrophages were among the most abundant immune cells in LUAD. Upon sub‐clustering of 32716 monocytes/macrophages, we discovered ten sub‐clusters (Figure **5**A) that belong to macrophages (C1‐4, C6, C9, C10) and monocytes (C5, C7, C8; Figure 5B,C). All these cell sub‐clusters were shared consistently across patients as well as within the IA, MIA, N, and PBMC samples, albeit with variations in their relative proportions (Figure 5D,E). The pseudotime analysis showed that all cells from PBMC were monocytes at the beginning of the trajectory path (Figure 5E,F). Monocytes/macrophages from N, MIA, and IA followed a similar transition trajectory in the early and middle stages, but the transition trajectory of macrophages from N and IA diverges in the late stages, while that of MIA is intermediate between the two mentioned above (Figure 5E,F), suggesting a dual evolution potential for MIA macrophages.

We found that mature macrophages from N, MIA and IA stages (C1‐3) highly expressed canonical marker genes of M1 and M2 macrophages (Figure 5G), indicating they displayed both M1 and M2 macrophage characteristics. However, GO analysis further revealed that the function of mature macrophages in the MIA stage (C2) was similar to that in the N stage (C1), but distinct from that in the IA stage (C3; Figure 5H). This indicates a closer similarity between mature macrophages in the N and MIA than between those in the MIA and IA.

We defined the TAM cluster based on their TAM scores, identifying C1, C2, C3, C4, and C6 as TAM clusters (Figure S5, Supporting Information). Importantly, we identified two crucial types of TAMs in the middle stages of macrophages (Figure 5G). One of these TAMs was marked by high expression of secreted phosphoprotein 1 (*SPP1*) and referred to as SPP1^+^ TAM (C4), while the other was marked by high expression of *CTSB* and referred to as CTSB^+^ TAM (C6). GO analysis showed that the function of SPP1^+^ TAM (S4) was primarily focused on antigen presentation, whereas CTSB^+^ TAM (S6) mainly activated the extracellular signal‐regulated kinase pathway (Figure 5H). The composition of monocytes/macrophages in PBMC differed significantly from N, MIA, and IA, comprising only monocytes. The proportion of SPP1^+^ TAM cells was higher in IA compared to N, but not greater in MIA than N. In contrast, the proportion of CTSB^+^ TAM cells in both IA and MIA were significantly higher than that in N, suggesting that CTSB^+^ TAM cells, instead of SPP1^+^ TAM cells, play a crucial role in MIA (Figure 5I). Consistent with these results, we found that the expression of *CTSB* was higher in the cells from MIA samples compared to that from IA sample in GSE189487 datasets^[^ ^14^ ^]^ (Figure 5J), while the expression of *SPP1* was lower in the cells from MIA samples compared to that from IA samples (Figure 5K). An integrated dataset of 719 LUAD patients corrected by Gyorffy^[^ ^20^ ^]^ showed that high expression levels of both CTSB and SPP1 significantly was associated with poor survival, highlighting the critical role of CTSB^+^ and SPP1^+^ TAM in the progression of LUAD (Figure 5L,M). Using IF, we measured the proportion of CTSB^+^ cells in CD68^+^ macrophages in the tissues of both discovery and validation cohorts, where we confirmed the abundance of a subset of CTSB^+^ CD68^+^ macrophages in MIA and IA, but not in N (Figure 5N,O). Overall, we found that CTSB^+^ TAM, but not SPP1^+^ TAM, was activated in MIA, which may play a crucial role in the progression of LUAD at the early stage.

### 2.5 CTSB^+^ TAMs Exhibited Potential Interactions with CD8^+^ Cytotoxic T Ceslls in MIA

**Figure 6:**
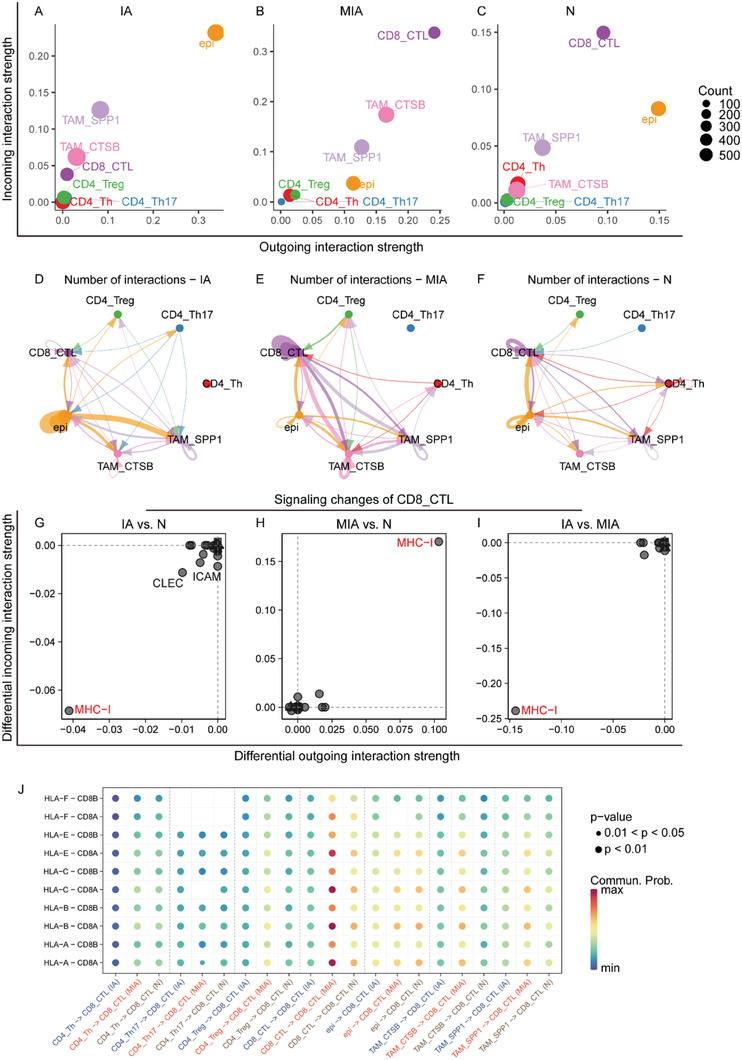
Intercellular interactions among different cells in N, MIA, and IA. The incoming and outgoing interaction strength of different cells in A) IA, B) MIA, and C) N. The number of interactions among different cells in D) IA, E) MIA, and F) N. The incoming and outgoing signaling changes of CD8^+^ CTL in G) IA versus N, H) MIA versus N, and I) IA versus MIA. J) Dot plot showing the MHC‐I interactions of different cells to CD8^+^ CTL. CTL, cytotoxic T lymphocyte; IA, invasive adenocarcinoma; L‐R, ligand‐receptor; MHC‐I, major histocompatibility complex class I; MIA, minimally invasive adenocarcinoma; N, adjacent normal tissue samples.

To characterize specific intercellular interactions among epithelial cells, subclusters of T cells and TAMs in MIA, we inferred communication networks using the ligand‐receptor interaction tool CellChat.^[^ ^21^ ^]^ The interaction strength of CD8^+^ CTL was robust in both MIA and N, while it was relatively weak in IA (Figure **6**A–C). In IA, malignant epithelial cells had the highest levels of incoming and outgoing interaction strength among all the cells, whereas in MIA, malignant epithelial cells showed a noticeable degree of outgoing interaction strength but limited incoming interaction strength (Figure 6A,B). More specifically, we found that both CTSB^+^ and SPP1^+^ TAMs had strong interaction in both IA and MIA. However, unlike in IA, the interaction strength of CTSB^+^ TAMs was stronger than that of SPP1^+^ TAM in MIA, suggesting that CTSB^+^ TAM play a more critical role in MIA (Figure 6A,B). The interactions within the epithelium itself and between SPP1^+^ TAMs and the epithelium were robust in IA and balanced among all cell types in N. Strong interactions were observed within CD8^+^ CTL, as well as between CTSB^+^ TAM and, to a lesser extent, SPP1^+^ TAM with CD8^+^ CTL (Figure 6D–F).

To decipher the specific signal changes associated with CD8^+^ CTL, we compared the interaction strength of each ligand‐receptor pair between CD8^+^ CTL and other cell types. Our findings showed that the interaction strength of major histocompatibility complex I (MHC‐I) was significantly stronger in MIA than in IA and N (Figure 6G–I). The ligands and receptors of MHC‐I expressed by CTSB^+^ TAM and SPP1^+^ TAM were exclusively more abundant in MIA than in IA and N (Figure 6J). Based on these findings, along with the increased interaction strength and interaction frequency between CTSB^+^ TAM and CD8^+^ CTL, coupled with the elevated presence of CTSB^+^ TAM in MIA, we propose that CTSB^+^ TAM may be responsible for the activation of CD8^+^ CTL in MIA.

## 3 Discussion

**Scheme 1:**
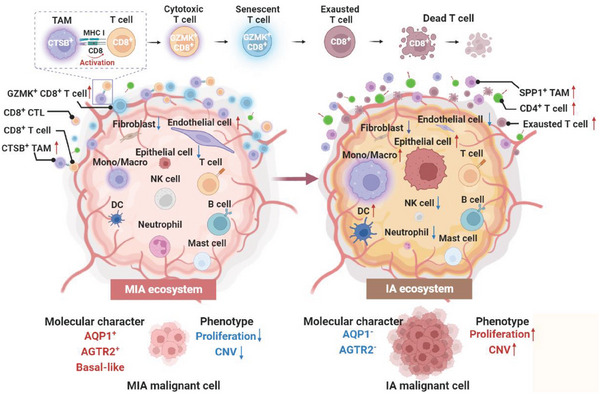
Molecular features and malignancy potential in lung minimally invasive adenocarcinoma. Differences in immune and tumor phenotypes were identified, including cell type and proportion, unique signatures of cancer cells, T cell and macrophages developmental trajectories, and the crosstalk among tumor and immune cells. Red arrows in the figure indicate an increase in cell numbers or enhanced functionality, while blue arrows represent a decrease in cell numbers or weakened functionality. The size of the legend for the major cell subgroups in the center of the image corresponds to their relative proportions in terms of cell numbers. *AGTR2*, angiotensin II receptor type 2; *AQP1*, aquaporin‐1; CNV, copy number variation; *CTSB*, cathepsin B; *GZMK*, granzyme K; IA, invasive adenocarcinoma; MHC I, major histocompatibility complex class I; MIA, minimally invasive adenocarcinoma; N, adjacent normal tissue samples; *SPP1*, secreted phosphoprotein 1.

Given the indolent growth pattern and good prognosis of MIA, it is essential to distinguish MIA from IA accurately and understand the mechanisms that maintain its indolent nature before making the surveillance and treatment strategies. Here, we performed a comprehensive single‐cell transcriptomic analysis to characterize and compare the tumor ecosystems between MIA and IA. We provided two hallmark signatures, namely AQP1 and AGTR2, of malignant cells in MIA, which in combination may serve as additional markers in distinction of MIA from IA. Our analysis revealed a distinct immune ecosystem in MIA, characterized by increased fractions of GZMK^+^ CD8^+^ T cells with dysfunctional cytotoxicity and CTSB^+^ TAMs with hyperactive cell communication. Transcriptomic profiling, augmented by L‐R based cell‐cell interaction analysis, suggested a potential crosstalk between CTSB^+^ TAM and CD8^+^ T cells in MIA, which may compromise antitumor immunity (Scheme **1**). Our study represents an essential step toward understanding how the immune environment shapes MIA and reveals the existence of active crosstalk between malignant cells and immune cells in MIA, facilitating the indolent nature of MIA tumor cells from anti‐tumor immunity.

It was found that the proliferative activity marker Ki‐67 expression was significantly lower in MIA than in IA.^[^ ^22^ ^]^ The low proliferative activity of MIA tumor cells may partly explain why MIA manifests as an indolent tumor. However, we also observed that the malignant cells in MIA featured a basal‐like molecular character. This suggests that MIA may not be as safe and harmless as we thought and should not be underestimated or ignored. Indeed, tumors with a basal molecular profile such as breast and bladder cancer are often more aggressive than other cancer subtypes.^[^ ^23^, ^24^ ^]^ This finding once again highlights the need to distinguish MIA and understand its prognosis. With the help of single‐cell transcriptomic technology, we identified two specific biomarkers, AQP1 and AGTR2, for MIA at the cellular resolution. We found that their expression was significantly increased in the MIA stage, while it gradually decreased as the tumor progressed. These findings were confirmed in another validation cohort, a publicly available scRNA‐seq data and TCGA database and supported by previous reports using other databases.^[^ ^25^, ^26^ ^]^ Additionally, we found in the TCGA database that the combination of these two biomarkers can effectively predict prognosis, even distinguishing patients with T1‐stage into good and poor prognostic groups.

The number and function of CD8^+^ T cells in LUAD decline with disease progression, but their status in MIA remains controversial.^[^ ^12^, ^13^, ^14^, ^15^, ^27^ ^]^ Here, we showed that MIA has a similar immune infiltration environment to normal tissue, but both the quantity and quality of CD8^+^ T cells decrease from MIA to IA. The anti‐tumor microenvironment of MIA may help restrain tumor growth and confer its indolent nature.^[^ ^28^ ^]^ Interestingly, we identify a subset of low‐cytotoxic and late‐stage CD8^+^ senescent T cells with high expression of *GZMK* in MIA. *GZMK* encodes granzyme K, which induces cell apoptosis and is typically associated with cytotoxic T cells.^[^ ^29^ ^]^ GZMK^+^ CD8^+^ T cells may also be a potential target to address age‐associated dysfunctions of the immune system.^[^ ^30^ ^]^ Given their characteristics of low cytotoxicity, late‐stage differentiation and highly expressed senescent markers, we suggested that these GZMK^+^ CD8^+^ T cells in MIA are senescence‐associated dysfunctional immune cells. Our results indicated that MIA has adequate T cell reserves in terms of numbers, but not functionality. Therefore, the anti‐tumor microenvironment of MIA is fragile and prone to disruption, which may trigger rapid tumor progression. MIA may be mistaken for an indolent tumor and its risk of tumor progression may be underestimated. Further studies are warranted to elucidate the functional status and diversity of different T cell subsets in MIA and their impact on tumor evolution and prognosis.

TAMs and T cells are functionally and spatiotemporally co‐dependent.^[^ ^31^ ^]^ Although it is well established that SPP1^+^ TAMs facilitate immune escape by inhibiting T cell activation and then affect the progression and metastasis of LUAD,^[^ ^32^, ^33^ ^]^ we identified a new subgroup of TAMs, CTSB^+^ TAMs, rather than SPP1^+^ TAMs, that play a major role in MIA. CTSB is a cysteine protease involved in inflammation, immunity, and tumor progression.^[^ ^34^ ^]^ In LUAD, CTSB expression correlates positively with immune cell infiltration and pro‐inflammatory cytokine expression.^[^ ^35^ ^]^ It has been observed that CTSB^+^ TAM and CD8^+^ CTL coexist spatially, forming an immune hub within the tumor. This suggests an interaction between these two cell types.^[^ ^36^ ^]^ In MIA, the increased presence of CTSB^+^ TAMs and their increased communication with CD8^+^ CTLs suggest that CTSB^+^ TAMs activate CD8^+^ CTLs, thus helping to maintain the indolent nature of MIA. However, the hyperinflammatory state can accelerate the process of T cell exhaustion.^[^ ^37^ ^]^ This may partly explain why there are so many senescent CD8^+^ T cells in MIA. The continuous accumulation of senescent CD8^+^ T cells ultimately leads to adverse prognostic outcomes. Indeed, it has been reported that CTSB^+^ TAMs play a vital role in promoting cancer metastasis and chemoresistance.^[^ ^38^ ^]^ Moreover, CTSB has been shown to be a negative prognostic biomarker and therapeutic target for some cancers, such as gliomas, hepatocellular carcinoma, and lung squamous cell carcinoma.^[^ ^34^, ^39^, ^40^ ^]^ In the present study, we found that CTSB was a negative prognostic biomarker for LUAD. Therefore, CTSB^+^ TAMs may have a dual role in MIA: beneficial in the short term but detrimental in the long term. Modulating the number or activity of CTSB^+^ TAMs may be a potential strategy for controlling MIA progression.

The analysis of peripheral blood for the detection and characterization of cancer has been widely employed.^[^ ^41^ ^]^ These blood‐based tests primarily rely on measuring cancer biomarkers, such as genetic and epigenetic alterations in circulating tumor DNA or proteins produced by cancer cells.^[^ ^41^, ^42^, ^43^ ^]^ An intriguing discovery from our study is the marked difference in the composition of immune cells between peripheral blood and tumor tissues, which may lead to inconsistent biological phenotypes between tumor cells surviving in the peripheral blood and those in the primary tumor site. While blood‐based tests have demonstrated promising clinical value, the underlying theoretical basis for this discrepancy requires further exploration in future investigations.

Our study has several limitations that are worth noting. First, the small tumor volume of MIA makes it challenging to obtain pure cancer cells, as the surgical resection tissue inevitably mixes with normal adjacent tissue. Consequently, the cell types and proportions estimated by scRNA‐seq data may contain some deviations. Second, our focus has been primarily on the subpopulation division and analysis of T cells and macrophages, centering on their cell communication interactions with tumor cells. While T cells and macrophages are prevalent immune cell types in MIA and IA, it is important not to ignore the roles of other immune and stromal cells. Therefore, further investigations are imperative to unravel the contributions of these cell types in subsequent research. Third, we did not conduct extensive in vivo and in vitro experimental validations of the interactions between CTSB^+^ TAM and CD8^+^ T cells. Future studies should determine if the spatial coordination of the induction of activation and exhaustion programs in CD8^+^ T cells is through CTSB^+^ TAM in the TME. Therefore, the clinical significance of our findings requires further investigation. Fourth, given the considerable patient heterogeneity, further studies will require an increased sample size to obtain more robust conclusions. Lastly, as a retrospective study, there may be some inherent biases.

Altogether, by using MIA and IA samples from patients with synchronous multiple primary LUAD, our study provides novel evidence of the tumor ecosystem connection and heterogeneity between MIA and IA. We found significant differences in the subtypes and quantities of cell populations within PBMCs when compared to both MIA and IA. Therefore, the feasibility of using PBMC samples for assessing LUAD in clinical settings still requires further investigation and evaluation. We identified differences in immune and tumor phenotypes, including cell type and proportion, unique signatures of cancer cells, T cell and macrophages developmental trajectories, and the crosstalk among tumor and immune cells. MIA tumor cells exhibited high expression of AQP1 and AGTR2 and a basal‐like molecular character. In MIA, CTSB^+^ TAMs might over‐activate CD8^+^ T cells, causing an enrichment of GZMK^+^ senescent CD8^+^ T cells, indicating the possibility of malignant progression underlying the indolent appearance of MIA. Our data can be a valuable resource, facilitating a deeper understanding of the mechanisms that maintain the indolent nature and prompt tumor progression of MIA, and assisting in developing more effective therapeutic targets and strategies for MIA patients.

## 4 Experimental Section

### Patients and Sample Collection

Three patients with synchronous multiple primary LUAD that included both MIA and IA were enrolled in this study as the Discovery cohort at the First Affiliated Hospital of Guangzhou Medical University. Fresh paired MIA and IA tumor samples, PBMCs, and adjacent normal tissue samples (at least 2 cm away from the matched IA tumor) were collected. Necrotic and hemorrhagic areas of the samples were removed, and the tumor tissues were divided into two parts: one piece (0.3 cm × 0.3 cm) was used for scRNA‐seq, while the other part was used for immunofluorescence. Blood samples were collected before surgery, and white blood cells were isolated and used for scRNA‐seq analysis. Formalin‐fixed paraffin‐embedded tissue blocks of MIA and IA were collected from another 69 LUAD patients (34 MIA and 35 IA patients) as the validation cohort. Additionally, 15 adjacent normal tissues from IA patients were also included in the validation cohort. All LUAD patients in both cohorts met the following criteria: i) pathological diagnosis of MIA or IA; ii) no previous history of other malignancies; iii) no previous anticancer treatment (such as chemotherapy, radiation therapy, or targeted therapy) before surgery; and iv) excluding pulmonary metastasis of the initial tumor per American College of Chest Physicians Guidelines.^[^ ^44^ ^]^ The detailed clinical and pathological information including age, gender, and tumor size is shown in Table S4 (Supporting Information). This study was approved by the Ethics Committee of the First Affiliated Hospital of Guangzhou Medical University (Guangzhou, China) (Ethics: Project identification code: 2020–89). All LUAD patients provided written informed consent.

### Sample Preparation and Sequencing

Tumor and normal tissue samples were immediately transported in ice‐cold Shbio's Tissue Preservation Kit (Shbio, Shanghai, China) after surgical resection. The tissues were rinsed with phosphate‐buffered saline (PBS; Thermo Fisher Scientific), minced into ≈1 mm cubes, and the cells were separated using Shbio's Cell Isolation Kit (Shbio). Cell counting was performed using a cell counter (Thermo Fisher Scientific). The RNA from single cells was barcoded using 10X Genomics Chromium Single Cell 3′ Library and Gel Bead Kit v3 and processed on a Chromium Single Cell Processor. Library construction followed the manufacturer's instructions (10X Genomics), and sequencing was performed on a NovaSeq 6000 sequencing system (Illumina).^[^ ^45^ ^]^

### Processing scRNA‐seq Data and Quality Control

The reads were processed using the Cell Ranger 3.0.1 pipeline with the recommended parameters. Using the STAR algorithm, the FASTQs were aligned generated from Illumina sequencing output to the human genome (version GRCh38).^[^ ^46^ ^]^ Then, gene‐barcode matrices were created for each sample by counting the unique molecular identifiers (UMIs) and filtering out any non‐cell associated barcodes. Finally, a gene‐barcode matrix was created with barcoded cells and gene expression counts. This output was imported into the Seurat (v. 3.0.2)^[^ ^47^ ^]^ R package to perform quality control and downstream analysis of our scRNA‐seq data. All functions were run with the default parameters unless otherwise specified. This study excluded cells with fewer than 200 or >6000 detected genes (with each gene having to be aligned with at least one UMI in at least three cells). The mitochondrial gene expression were calculated using the Seurat package's percentage feature set function. To eliminate low activity cells, any cells were excluded with >10% mitochondrial gene expression. The data were normalized using the normalize data function in the Seurat package to extract a subset of variable genes. While controlling for the strong relationship between variability and average expression, variable genes were identified. Finally, data were integrated from different samples by identifying “anchors” between datasets using the find integration anchors and integrate data functions in the Seurat package.

### Unsupervised Clustering and Biomarker Identification

Principal component analysis (PCA) was performed and reduced the data to the top 30 PCA components after scaling the data. The clusters were visualized on a 2D map produced with Umap^[^ ^40^ ^]^ and identified cell types and subtypes by nonlinear dimensional reduction. Cells were clustered using graph‐based clustering of the PCA reduced data with the Louvain Method^[^ ^48^ ^]^ after computing a shared nearest neighbor graph.^[^ ^49^ ^]^ To identify the markers for each cluster, a differential expression of each cluster was performed against all other clusters, identifying positive markers for each cluster. Cells were exatracted from broad cell types (epithelial cells, T cells, and monocyte/macrophage clusters) and re‐clustered them to further analyze the sub‐clusters in each cell type. This study used the SingleR (v. 1.4.1)^[^ ^50^ ^]^ package of R and known marker genes to identify cell type.

### Analysis of Differential Gene Expression and Pathway

This study performed pairwise comparison of gene expression among IA, MIA, and N using the “FindMarkers” function from the Seurat package ((Wilcoxon Rank Sum test). Genes were filtered with |log_2_FC | ≥ 0.5, *P_adjust_* ≤ 0.05 to generate the DEG lists. A ternary diagram was then generated for the DEGs using the ggtern package.^[^ ^51^ ^]^ This study conducted enrichment analysis with the clusterProfiler^[^ ^52^ ^]^ (v. 3.14.0) package of R with databases from the KEGG with the DEG lists as input. The significance of pathways was set at *q* ≤ 0.05 and *P_adjust_* ≤ 0.05.

### Copy Number Variations Estimation and Identification of Malignant Cells

To identify malignant cells with clonal large‐scale chromosomal CNVs, the inferCNV R package^[^ ^53^ ^]^ was used to infer the genetic profiles of each cell based on the average expression of large genes sets (101 genes) in each chromosomal region of the tumor genome compared to normal cells. All epithelial cells were designated as the interrogation group and T and NK cells as putative non‐malignant cells as control. The other parameters were set to the default values. This study considered the inferCNV level along with the expression of malignant cell markers to determine the malignant subclusters.

### Trajectory Inferencse and Pseudotime Analysis

Monocle3,^[^ ^54^ ^]^ slingshot^[^ ^18^ ^]^ and scTour^[^ ^19^ ^]^ were used for the trajectory inference and pseudotime analysis. For Monocle3, a principal graph was created by using the “learn_graph” function and selected the root position programmatically by using a helper function provided by the author of Monocle3. Briefly, a group of “early cells” was designated, which, for epithelial cells, these were cells originating from normal tissue, and for T cells and macrophages, these were cells from PBMC. Subsequently, this study identified the node that was predominantly occupied by “early cells” and designated it as the root node. Pseudotime values were then assigned to each cell with “order_cells”. Genes with differential expression were identified along the inferred trajectories with the “top_marker” function. After identifying these genes, a gene ontology (GO) analysis was considered to further understand the differences. Finally, gene expression and pathway score patterns were visualized and analyzed with the “plot_gene_in_pseudotime” function. For slingshot, this study utilized the parameters and code recommended by the authors and determined the starting point through unsupervised clustering. For scTour, this study utilized the parameters and code recommended by the author.

### Correlation to TCGA Data and Survival Analysis

The TCGA‐LUAD RNA‐seq gene expression and clinical dataset were downloaded from TCGA Data Portal (https://tcga‐data.nci.nih.gov/tcga/). The gene expression data was normalized by applying log_2_(FPKM+1). To compare the difference in survival between MIA and other stages of LUAD, the TCGA samples were grouped based on their tumor stage. Taking advantage of a higher number of patients, the difference were compared in survival between LUAD patients with different gene expression levels using an online survival analysis software. Kaplan–Meier survival curves were plotted to visualize differences in survival time and used the log‐rank p values reported by the Cox regression models implemented in the R package “survival” to determine the statistical significance.

### Definition of Signature Scores of Cells

Cell scores were used to evaluate the degree to which individual cells expressed a certain predefined expression gene set. The cell scores were initially based on the average expression of the genes from a predefined gene set in the respective cell.^[^ ^49^ ^]^ The AddModuleScore function in Seurat was used to implement the method with default settings. Genes with unavailable expression data were excluded from calculations of gene signature scores. The gene sets used in this study are list in Table S5 (Supporting Information).

### Cell–Cell Interaction Network Analysis

To analyze cell‐cell interactions between different cell types, CellChat^[^ ^21^ ^]^ was used to identify significant ligand‐receptor pairs within normal, MIA, and IA samples. The cell type specific receptor‐ligand interactions between cell types were identified based on the specific expression of a receptor by one cell type and a ligand by another cell type. The interaction score refers to the total mean of the individual ligand‐receptor partner average expression values in the corresponding interacting pairs of cell types. The visualizations were created following the R‐package's instructions.

### Multiplex Immunofluorescence

Multiplex immunofluorescence staining of tissues from the discovery and validation cohorts were performed using TSAPLus multiple fluorescence staining kit (Cat# G1236, Servicebio) according to manufacturer's instructions. The slides were used for triple staining of AQP1 (Cat# 66 805, Proteintech), AGTR2 (Cat# bs‐2133r, Bioss), and EpCAM (Cat# GB12274, Servicebio) antibodies, double staining of GZMK (Cat# 67 272, Proteintech) and CD8 (Cat# 66 868, Proteintech) antibodies, and double staining of CTSB (Cat# 12 216, Proteintech) and CD68 (Cat# YM3050, ImmunoWay) antibodies. Nuclei were stained with 4′,6‐diamidino‐2‐phenylin‐ dole (DAPI) solution (Cat# G1012, Servicebio) after all the antigens above had been labeled. The complete list of the antibodies is shown in Table S6 (Supporting Information). The stained slides were scanned to obtain multispectral images using the Pannoramic MIDI (3DHISTECH), which captured the fluorescent spectra at 420, 520, 620, and 700 nm with identical exposure times, respectively. For each slide, five fields of tumoral area were selected for image capture and quantification. The fluorescence intensity of each image was measured by using ImageJ 1.52 v (National Institutes of Health, Bethesda, MD, USA).

### Statistical Analyses

Statistical analyses were performed in R^[^ ^55^ ^]^ (v. 4.0.2) and SPSS 22.0 (IBM, Armonk, NY). Comparisons of gene expression or signature scores among different groups of cells were performed using a one‐way analysis of variance (ANOVA) followed by a Tukey's multiple comparisons test, unless otherwise indicated. Statistical tests were two‐sided, and a *p*‐value of < 0.05 was considered significant.

## Conflict of Interest

The authors declare no conflict of interest.

## Author Contributions

X.Z., B.L., and Y.H. contributed equally to this work as co‐first authors. X.Z., B.L., and Y.H. performed conceptualization, methodology, visualization, formal analysis, wrote the original draft. H.M., Z.L., J.D., L.Z., Y.Z., B.W., X.L., X.C., and W.L. performed visualization and data curation. X.Z. and X.Y. performed funding acquisition, and acquired resources. G.Y. and Z.‐S.C. wrote reviews and edited the original manuscript. Z.H. performed conceptualization, acquired resources, funding acquisition, project administration, wrote reviews, and edited the original manuscript.

## Supporting information

Supporting Information

## Data Availability

The data that support the findings of this study are available from the corresponding author upon reasonable request.
