# Supplementary material for: Behind the Indolent Facade: Uncovering the Molecular Features and Malignancy Potential in Lung Minimally Invasive Adenocarcinoma by Single‐Cell Transcriptomics
Source: Adv Sci (Weinh). 2023 Nov 22;10(36):2303753. doi: 10.1002/advs.202303753 (PMC10754125; doi:10.1002/advs.202303753)
Supplement: Supplementary file 1 — Supporting Information [file ADVS-10-2303753-s001.pdf]

ADVANCED SCIENCE
Open Access

Supporting Information

for *Adv. Sci.*, DOI 10.1002/advs.202303753

Behind the Indolent Facade: Uncovering the Molecular Features and Malignancy Potential in Lung Minimally Invasive Adenocarcinoma by Single-Cell Transcriptomics

*Xin Zhang, Boxuan Liang, Yuji Huang, Hao Meng, Zhiming Li, Jiaxin Du, Lang Zhou, Yizhou Zhong, Bo Wang, Xi Lin, Guangchuang Yu, Xuewei Chen, Weixiang Lu, Zhe-Sheng Chen, Xingfen Yang and Zhenlie Huang**

Supporting Information

Table S1. Clinical and pathological information of patients in discovery cohort

Patient ID	Age	Gender	Number of lesions	Tumor type	Size (cm)	Affected Lobe	Distant metastasis	Driver gene mutation
No.1	73	Female	5	IA/IA/MIA/MIA/MIA	3.5[a]/1.3/0.5[a]/0.4[a]/0.2	Left upper, right upper	No	KRAS_G12R (1.42%) and EGFR E746_A750de (146.48%)[b]
No.2	73	Male	3	IA/MIA/MIA	4.7[a]/0.7/0.5	Left upper, left lower, right upper	No	KRAS_G12V (21.2%)[b]
No.3	67	Male	5	IA/IA/IA/IA/MIA	2.6[a]/2.2[a]/1.8/1.3/0.9[a]	Right upper, right middle, right lower	No	EGFR_S768I (31.6%) and EGFR_G719C (30.1%)[b]

a: Selected tumor sample for scRNA-seq;

b: Driver gene mutation for the selected IA sample.

Table S2. Quality control of scRNA data

Sample	Cell number	Mean reads per cell	Median genes per cell	Total genes detected	Median UMI counts per cell
IA_No.1	15,625	38,696	1,757	25,301	6,092
IA_No.2	11,844	50,299	2,202	25,050	7,284
IA1_No.3	9,736	65,121	3,862	23,418	16,520
IA2_No.3	13,364	42,375	2,945	24,328	10,105
MIA_No.3	12,471	47,829	1,426	24,250	2,995
MIA1_No.1	8,789	64,857	1,540	23,118	4,244
MIA2_No.1	12,007	51,055	1,612	23,733	4,398
N_No.1	15,508	39,037	1,455	24,962	4,149
N_No.2	10,686	53,935	1,534	23,864	4,503
N_No.3	15,088	39,599	1,737	25,152	4,695
PBMC_No.1	10,324	63,667	1,493	21,496	4,969
PBMC_No.2	9,607	59,907	1,385	21,290	3,835
PBMC_No.3	11,398	51,921	1,622	21,616	5,797
Mean	12,034	51,408	1,890	23,660	6,122
Min	8,789	38,696	1,385	21,290	2,995
Max	15,625	65,121	3,862	25,301	16,520
Median	11,844	51,055	1,612	23,864	4,695

Table S3. The absolute cell numbers of non-immune and immune cells in each type of tissue

Cell type	N	MIA	IA	PBMC
Immune cells				
Mono/Macro	9950	7910	11915	3341
NK cell	7767	5892	758	5627
T cell	4150	4080	4420	13429
B cell	338	136	560	1474
Neutrophil	732	186	69	513
Mast cell	752	372	127	8
DC	316	416	795	61
Non immune cells				
Epithelial	3560	801	14291	23
Fibroblast	2261	1135	236	7
Endothelial	1118	6620	289	29

Table S4. Clinical pathological feature of validation cohort

**Characteristic**	**MIA (*n* = 34)**	**IA (*n* = 35)**
Age (mean ± SD), yr	54.9 ± 8.7	58.7 ± 11.6
Sex		
Female, *n* (%)	38.2%	42.9%
Male, *n* (%)	61.8%	57.1%
Largest diameter (mean ± SD), cm	0.4 ± 0.1	2.8 ± 0.9

Table S5. Gene sets used in this study

**Cytotoxic (Related to Fig. 4J)**	**Exhaustion (Related to Fig. 4K)**
GZMA	LAG3
GNLY	TIGIT
PRF1	HAVCR2
GZMB	CTLA4
GZMK	PDCD1
IFNG	
NKG7	
GZMH	

Table S6. List of antibodies for IF

Primary antibody	Manufacturer	Catalog	Dilution	Second antibody	Manufacturer	Catalog	Dilution
EpCAM	Proteintech	GB15274	1:2000	HRP-labeled Goat Anti-Mouse IgG	Servicebio	GB23301	1:500
AQP1	Proteintech	66805-1-IG	1:200	Cy3-labeled Goat Anti-Mouse IgG	Servicebio	GB21301	1:300
AGTR2	bioss	BS-2133R	1:200	Cy5-labeled Goat anti-Rabbit IgG	Servicebio	GB27303	1:400
CD8	Proteintech	66868-1-IG	1:3000	HRP-labeled Goat Anti-Mouse IgG	Servicebio	GB23301	1:500
GZMK	Proteintech	67272-1-IG	1:500	Alexa Fluor 488-labeled Goat Anti-Mouse IgG	Servicebio	GB25301	1:400
CTSB	Proteintech	12216-1-AP	1:500	Alexa Fluor 488-labeled Goat anti-Rabbit IgG	Servicebio	GB25303	1:400
CD68	Servicebio	GB113150	1:3000	HRP-labeled Goat anti-Rabbit IgG	Servicebio	GB23303	1:500

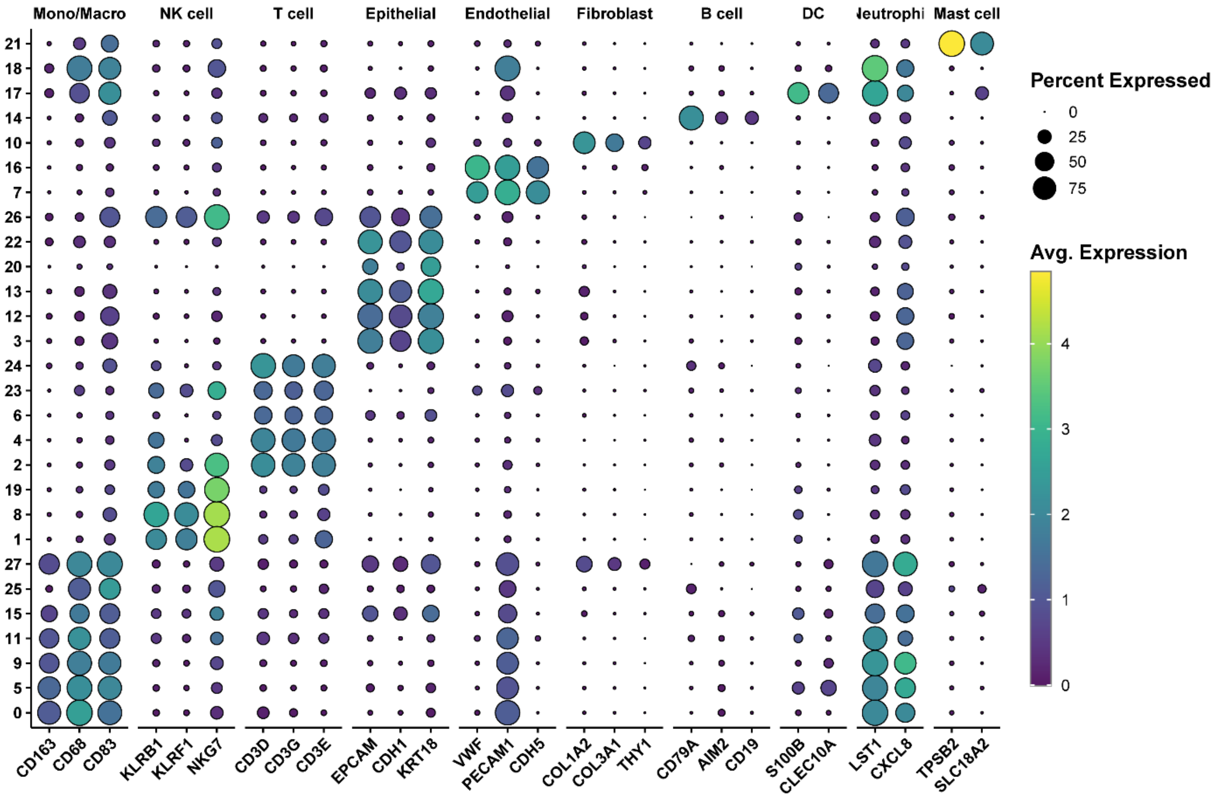

Figure S1. Heatmap indicating the expression of canonical marker genes in each cell type.

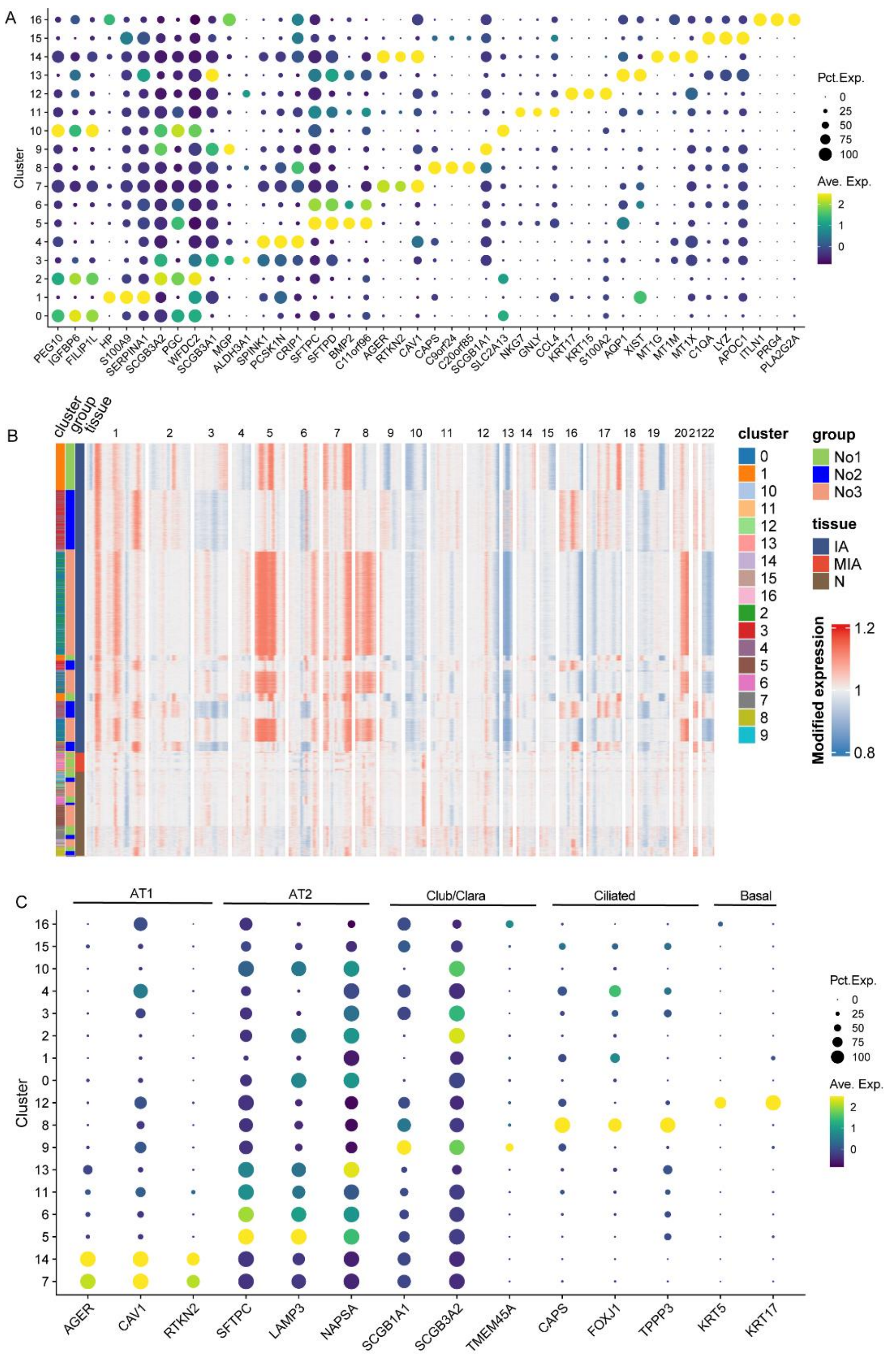

Figure S2. A) Heatmap indicating the top positive marker gene in each cluster of epithelial cells. B) Heatmap showing large-scale inferCNVs in sample type order for individual epithelial cells in N, MIA, and IA. C) Heatmap indicating the expression of canonical marker genes in normal cell types of epithelial cells. CNV, copy number variant; IA, invasive adenocarcinoma; MIA, minimally invasive adenocarcinoma; N, adjacent normal tissue samples.

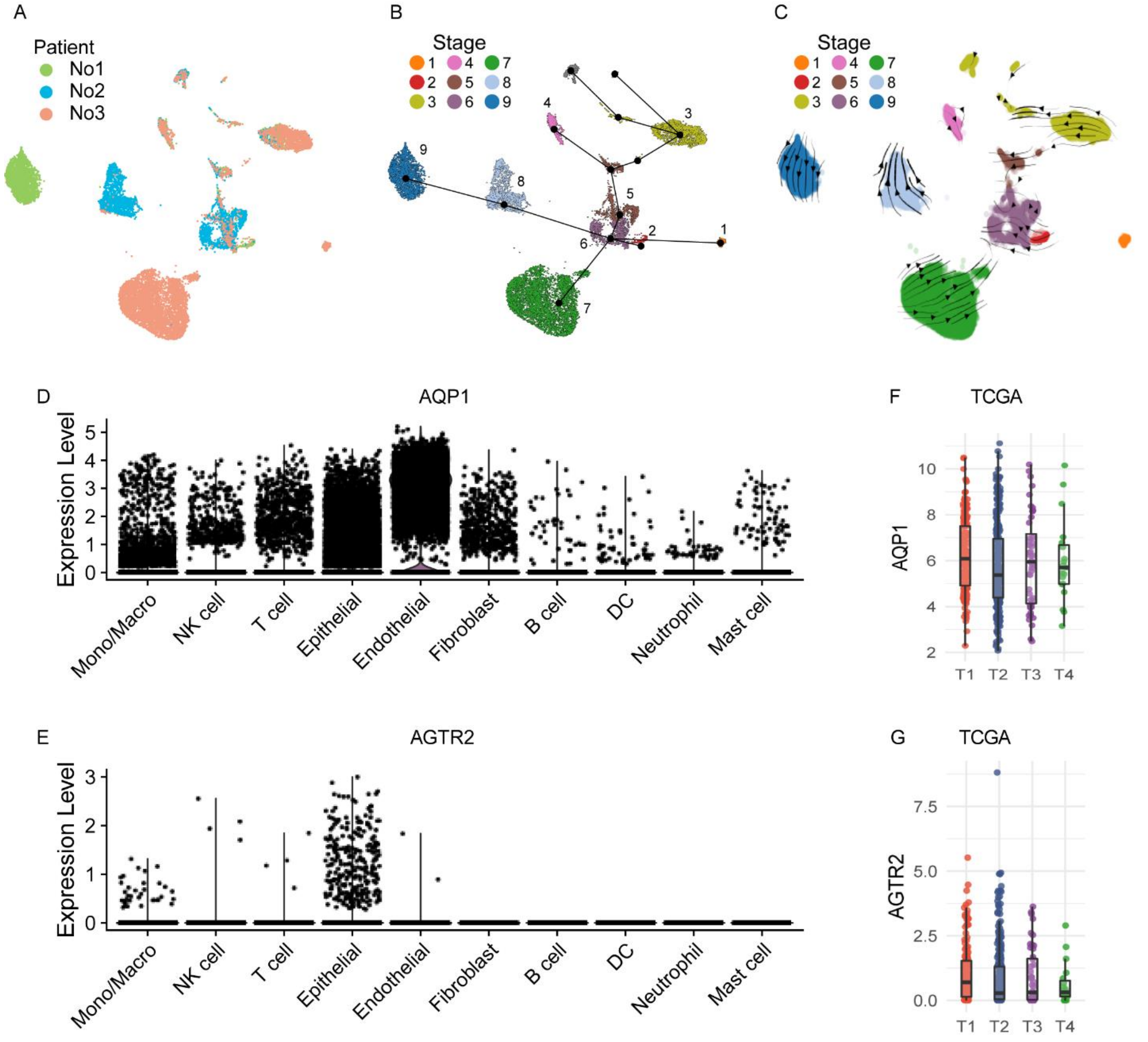

Figure S3. A) UMAP plot displaying the pseudotime-ordered analysis of epithelial cells from N, MIA, and IA samples, cells are colored by patients. B) UMAP plot displaying the state trajectories inferred by slingshot. C) UMAP plot displaying the developmental dynamics inferred by scTour. Violin plot illustrating the expression of D) *AQP1* and E) *AGTR2* in each type of cells in our scRNA-seq data. Bar plots indicating the expression of F) *AQP1* and G) *AGTR2* in each cancer stage in TCGA-LUAD dataset. *AGTR2*, angiotensin II receptor type 2; *AQP1*, aquaporin-1; IA, invasive adenocarcinoma; LUAD, lung adenocarcinoma; MIA, minimally invasive adenocarcinoma; N, adjacent normal tissue samples; scRNA-seq, single-cell RNA sequencing; TCGA, The Cancer Genome Atlas.

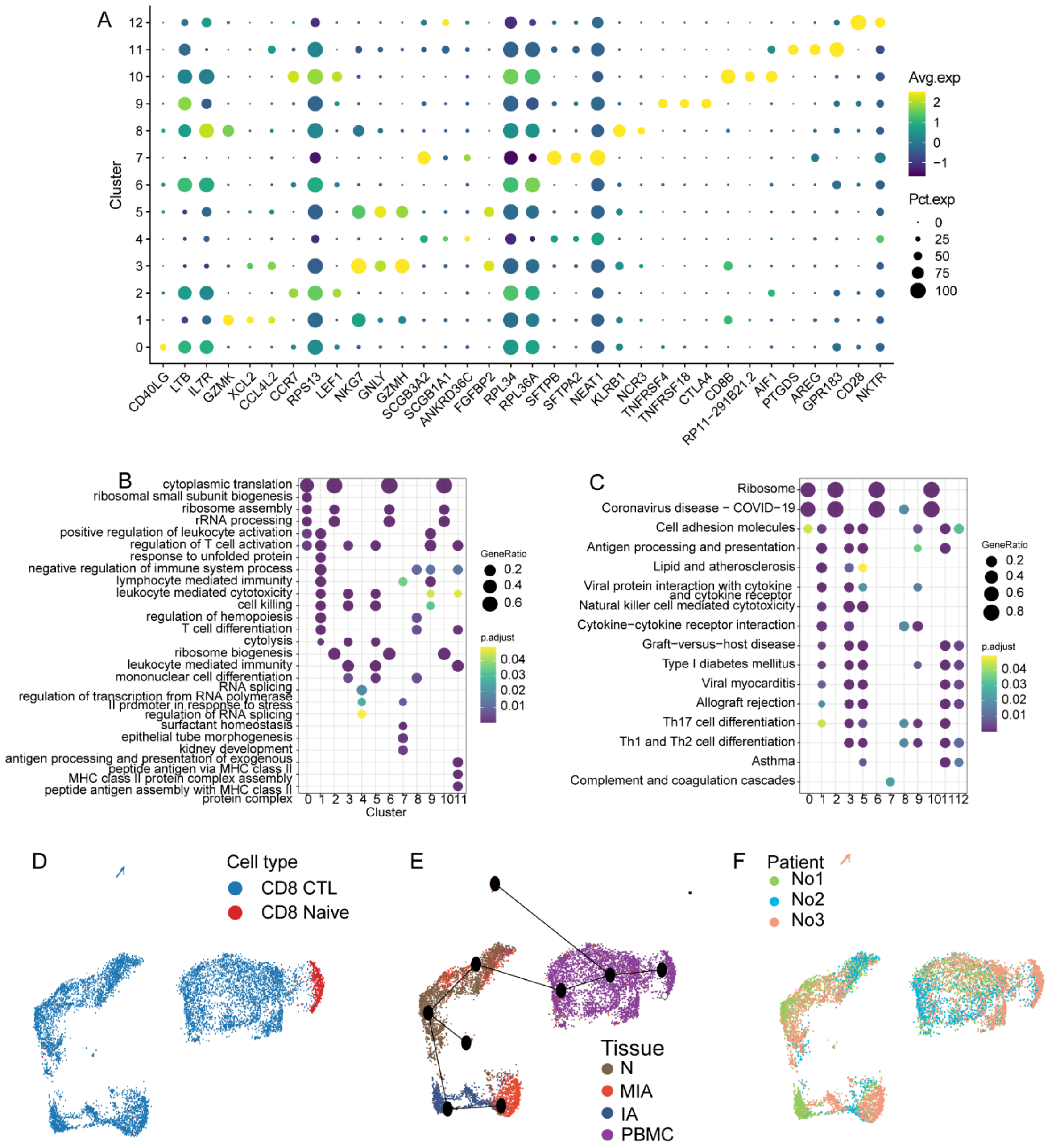

Figure S4. A) Heatmap indicating the top positive marker gene in each cluster of T cells. Enrichment of B) GO and C) KEGG pathways for high expressed DEGs in N, MIA, and IA tissues. D) UMAP plot displaying the pseudotime-ordered analysis of $CD8^+$ T cells from N, MIA, and IA samples, cells are colored by cell types. E) UMAP plot displaying the state trajectories inferred by slingshot. F) UMAP plot displaying the pseudotime-ordered analysis of $CD8^+$ T cells from N, MIA, and IA samples, cells are colored by patients. GO, Gene Ontology; IA, invasive adenocarcinoma; KEGG, Kyoto Encyclopedia of Genes and Genomes; LUAD, lung adenocarcinoma; MIA, minimally invasive adenocarcinoma; N, adjacent normal tissue samples; PBMC, peripheral blood mononuclear cell.

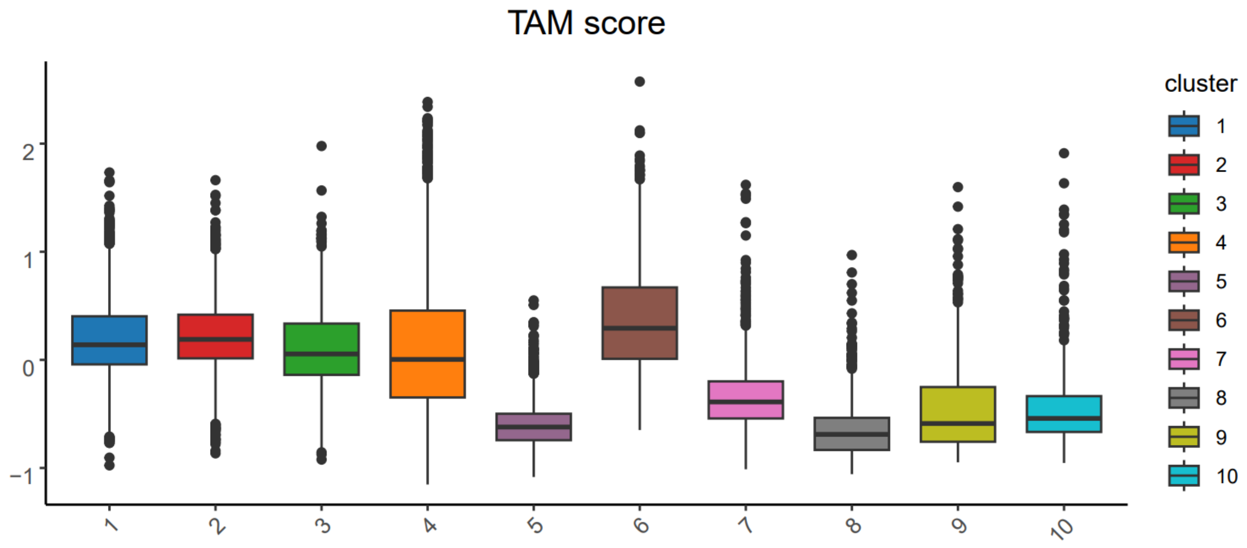

Figure S5. TAM score of each monocyte/macrophage cluster.
